# Kynurenine Pathway after Kidney Transplantation: Friend or Foe?

**DOI:** 10.3390/ijms25189940

**Published:** 2024-09-14

**Authors:** Izabela Zakrocka, Ewa M. Urbańska, Wojciech Załuska, Andreas Kronbichler

**Affiliations:** 1 Department of Nephrology, Medical University of Lublin, 20-093 Lublin, Poland; izabela.zakrocka@umlub.pl (I.Z.); wojciech.zaluska@umlub.pl (W.Z.); 2 Department of Experimental and Clinical Pharmacology, Medical University of Lublin, 20-093 Lublin, Poland; ewa.urbanska@umlub.pl; 3 Department of Internal Medicine IV, Nephrology and Hypertension, Medical University Innsbruck, 6020 Innsbruck, Austria; 4 Department of Health, Medicine and Caring Sciences, Linköping University, 581 83 Linköping, Sweden

**Keywords:** kidney, transplantation, graft, rejection, immunosuppression, kynurenine, kynurenic acid, tryptophan, infection, cancer

## Abstract

Kidney transplantation significantly improves the survival of patients with end-stage kidney disease (ESKD) compared to other forms of kidney replacement therapy. However, kidney transplant recipients’ outcomes are not fully satisfactory due to increased risk of cardiovascular diseases, infections, and malignancies. Immune-related complications remain the biggest challenge in the management of kidney graft recipients. Despite the broad spectrum of immunosuppressive agents available and more detailed methods used to monitor their effectiveness, chronic allograft nephropathy remains the most common cause of kidney graft rejection. The kynurenine (KYN) pathway is the main route of tryptophan (Trp) degradation, resulting in the production of a plethora of substances with ambiguous properties. Conversion of Trp to KYN by the enzyme indoleamine 2,3-dioxygenase (IDO) is the rate-limiting step determining the formation of the next agents from the KYN pathway. IDO activity, as well as the production of subsequent metabolites of the pathway, is highly dependent on the balance between pro- and anti-inflammatory conditions. Moreover, KYN pathway products themselves possess immunomodulating properties, e.g., modify the activity of IDO and control other immune-related processes. KYN metabolites were widely studied in neurological disorders but recently gained the attention of researchers in the context of immune-mediated diseases. Evidence that this route of Trp degradation may represent a peripheral tolerogenic pathway with significant implications for transplantation further fueled this interest. Our review aimed to present recent knowledge about the role of the KYN pathway in the pathogenesis, diagnosis, monitoring, and treatment of kidney transplant recipients’ complications.

## 1. Introduction

Kidney diseases represent a broad spectrum of disorders with various etiologies and clinical presentations. Although the number of individuals worldwide affected by kidney diseases is estimated at 850 million, their true prevalence seems to be largely underestimated [1]. It was suggested that kidney disorders are one of the most common healthcare problems, as the number of people affected by kidney diseases is significantly larger than patients with diabetes or individuals infected by the human immunodeficiency virus (HIV) [1]. Chronic kidney disease (CKD), the predominant kidney disorder with a significant impact on health outcomes, often diagnosed late or unrecognized, is expected to be the 5th leading cause of death by 2040 [2]. Patients with CKD are at exceptionally high risk of cardiovascular events, largely dependent on preexisting comorbidities and the presence of specific risk factors [3,4]. The incidence of cardiovascular events was reported to be even higher in advanced CKD, namely end-stage kidney disease (ESKD), resulting in a 5-year survival rate in patients receiving kidney replacement therapy (KRT) of only 48% in Europe and 41% in the United States of America [5]. According to the European Renal Association Registry, the unadjusted incidence of KRT in Europe in 2021 was 145 per million, whereas the number of kidney transplantations performed per million was estimated at 37 [6]. Among KRT methods, kidney transplantation represents the gold standard of treatment for ESKD, resulting in a substantial improvement in patients’ 5-year survival, ranging from 85.8% (deceased donor) to 94.2% (live donor) [7]. The unadjusted 5-year graft survival was shown to be lower, 77.0% in deceased donor transplantations vs. 88.3% in live donor kidney transplantations [7]. In Europe, despite a lower transplant rate than in America, more ESKD patients live with a functioning graft (47% vs. 32%, respectively). This can still be considered unsatisfactory [8]. Despite the improvements in surgical techniques, organ preservation methods, and immunosuppressive modalities, patient and graft survival are limited, mainly due to immunological damage of the transplanted organs, as well as cardiovascular, infectious, and oncological complications [9,10]. Searching for reliable and well-validated biomarkers is of special importance in kidney transplant recipients’ care [11]. Proposed biomarkers, including plasma donor-derived cell-free DNA (dd-cfDNA), blood Torque teno virus copy numbers, or urinary chemokine tests, are promising noninvasive methods of graft function evaluation already under investigation [12,13]. However, the increasing number of kidney transplant recipients necessitates the search for novel strategies for the diagnosis and treatment of transplant-related complications.

Tryptophan (Trp) is one of the eight essential amino acids. Yet, only 1% of its pool is used for protein synthesis [14]. The remaining 99% of available Trp is metabolized into biologically active substances, including serotonin (1–2%), and several molecules formed along the kynurenine (KYN) pathway [15]. The KYN pathway, a major route of Trp degradation, has been extensively studied in the context of neurological [16,17], inflammatory [18], oncological [19], kidney [20,21], cardiovascular [22,23], and metabolic disorders [24,25]. The functional link between Trp metabolism and organ transplant complications received increasing attention during the last few years. The Trp-KYN pathway was implicated in the host defense against pathogens and in peripheral mechanisms of tolerance induction, mainly through its impact on T cell activity [26]. 

In this review, we discuss the putative role of the KYN pathway in immunological control after kidney transplantation and in the risk of infectious, oncological, and cardiovascular complications. Additionally, diagnostic and therapeutic implications for kidney transplant recipients’ management are highlighted. 

## 2. The Kynurenine (KYN) Pathway

**Figure 1:**
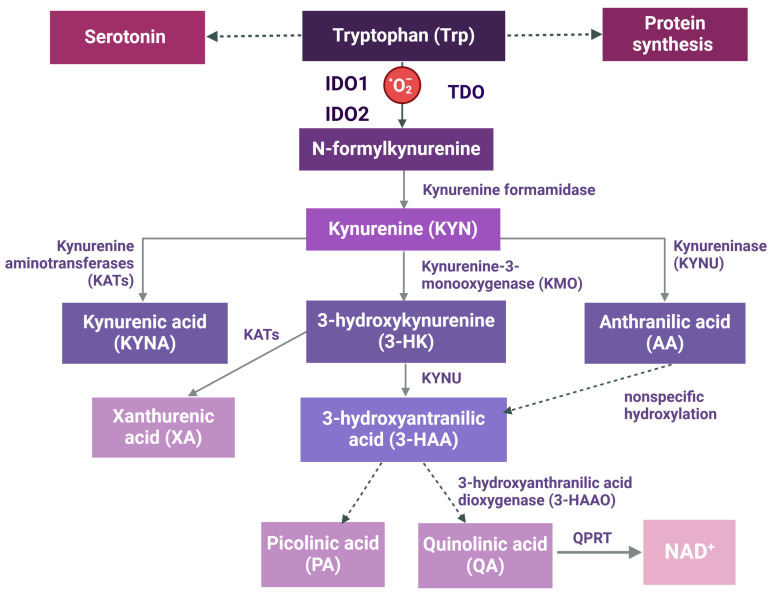
The kynurenine pathway. 3-HAA, 3-hydroxyanthranilic acid; 3-HK, 3-hydroxykynurenine; AA, anthranilic acid; 3-HAAO, 3-hydroxyanthranilic acid dioxygenase; IDO1, indoleamine-2,3-dioxygenase 1; IDO2, indoleamine-2,3-dioxygenase 2; KATs, kynurenine aminotransferase; KMO, kynurenine-3-monooxygenase; KYN, kynurenine; KYNA, kynurenic acid; KYNU, kynureninase; NAD^+^, nicotinamide adenine dinucleotide; PA, picolinic acid; QA, quinolinic acid; QPRT, quinolinic acid phosphoribosyltransferase; TDO, tryptophan-2,3-dioxygenase; Trp, tryptophan; XA, xanthurenic acid. Created with https://www.BioRender.com (accessed on 1 August 2024).

Trp degradation begins with a rate-limiting step, which is catalyzed by 3 enzymes: indoleamine-2,3-dioxygenase 1 (IDO1), indoleamine-2,3-dioxygenase 2 (IDO2), or tryptophan-2,3-dioxygenase (TDO) (Figure 1). 

All Trp-degrading enzymes are hemoproteins [27]. TDO is exclusively expressed in the liver and central nervous system and controls the entrance of Trp into the KYN pathway under physiological conditions [28]. Due to its low affinity for Trp, TDO is active when Trp concentrations exceed those required for protein or serotonin synthesis, indicating the role of TDO in the prevention of Trp accumulation [15]. Several factors, including Trp [29], glucocorticoids (GCs), and glucagon [30,31], were shown to upregulate TDO activity. In contrast to TDO, IDO is an inducible enzyme widely distributed in various cells; in immune cells, it includes monocytes, macrophages [32], and dendritic cells (DCs) [33]. IDO gene transcription is mainly stimulated by interferon-γ (IFN-γ) [34], tumor necrosis factor-α (TNF-α), interleukin-1 (IL)-1 [35], and toll-like receptors (TLRs) activated by lipopolysaccharide (LPS) [36]. However, IDO expression should not be considered equal to IDO’s activity, since some DCs may constitutively express IDO and require stimulatory signals (i.e., superoxide) for posttranslational heme modification to activate IDO [37]. During the activation of IDO by immunological stimuli, the activity of TDO is substantially suppressed, and IDO remains the main enzyme responsible for Trp breakdown [38]. Additionally, IDO has a lower capacity with a significantly higher affinity for Trp compared to TDO [39]. Two distinct isoforms of IDO have been found in humans. Whereas IDO1 seems to play a major role in Trp depletion, resulting in antimicrobial defense and T cell suppression, and most available data regarding IDO are related to IDO1, the role of IDO2 is much less explored [40]. Several studies suggested IDO2 involvement in oncogenesis [41] through the inhibition of immune response to tumor cells [42]. However, other Trp-degrading enzymes, TDO and IDO1, have also been found on cancer cells, pointing to their role in facilitating the immune escape of tumors [28]. 

After the transformation of Trp to N-formylkynurenine (N-formyl KYN) by IDO or TDO, the next step entails the formation of KYN, which later can be converted into three types of metabolites. KYN is preferentially metabolized into 3-hydroxykynurenine (3-HK) by kynurenine-3-monooxygenase (KMO) and then by kynureninase (KYNU) into 3-hydroxyanthranilic acid (3-HAA), which is a substrate for picolinic acid (PA) or quinolinic acid (QA) production, with nicotinamide adenine dinucleotide (NAD^+^) as a final product [43]. Although some data show that most Trp metabolites from this branch, especially 3-HK, 3-HAA, and QA, act as antioxidants [44], the majority of reports indicate that they exert pro-oxidant activity [45,46,47] and thus may evoke cell death [48]. IDO/TDO and KMO enzymes are crucial to the control of Trp degradation and its metabolic effects.

Kynurenic acid (KYNA), synthesized from KYN by kynurenine aminotransferases (KATs), is a broad-spectrum antagonist of all types of ionotropic glutamate (GLU) receptors of N-methyl-D-aspartate (NMDA) [49], alpha-amino-3-hydroxy-5-methyl-4-isoxazolepropionic acid (AMPA), and kainate [50] type. Neuroprotective, antiepileptic, and neuromodulatory effects of KYNA are well described, and the possible impact of KYNA on brain disorders is broadly accepted [51,52,53,54]. KYNA was also shown to inhibit α7 nicotinic acetylcholine receptors [55] and to act as a ligand for aryl hydrocarbon receptor (AhR) [56] and G protein-coupled receptor 35 (GPR35), which regulate the immune response [57]. Consequently, through the AhR- [58] and GPR35-mediated processes, KYNA may act as an immunosuppressive agent [59] and a guardian of gut microbiota homeostasis, linking the immune system with microbiota and metabolic disorders [60,61]. 

The third branch of the KYN pathway, yielding anthranilic acid (AA), is the least studied route of Trp metabolism. Since AA can be converted through nonspecific hydroxylation into 3-HAA (Figure 1), it becomes a precursor for neuroactive products. Although AA itself presented ambiguous properties in the context of neurological disorders, a complex interplay between AA and the gut-brain axis was recently highlighted [62].

Trp and its metabolites have been extensively studied in CKD patients. KYN pathway activation, presented as IDO induction, and high serum KYN, KYNA, and QA levels correlate with CKD severity and inflammatory parameters, high-sensitivity C-reactive protein (hsCRP), and soluble TNF-receptor-1 (sTNFR-1) concentration, independently of serum creatinine level [63]. Additionally, KYN and its metabolites have been linked to oxidative stress [64], endothelial dysfunction [65], cognitive impairment [66], and incident cardiovascular disease in CKD patients [67,68]. Although products of Trp degradation can accumulate in the body secondary to impaired glomerular filtration, their level, especially in the serum, was shown to tightly correlate with immune system activation and possibly to predict kidney damage and its progression. The measurement of serum IDO activity together with the Naples prognostic score has been recently proposed as a useful tool in early CKD diagnosis [69]. 

Importantly, Trp is present at a 2 mmol/L concentration in one of the most popular preservation solutions, a histidine-tryptophan-ketoglutarate (HTK) solution, mainly because of its antioxidant and membrane-stabilizing potential [14]. HTK, invented by Bretschneider, was shown to be a comparable preservation solution when preservation times were shorter and when standard criteria donor organs were implanted [70,71]. Modified HTK solution, a HTK-N solution, was shown to lower endothelial graft damage, although observations in kidney graft recipients are limited [72]. On the other hand, Trp deprivation is suggested to induce resistance to hypoxia and prevent ischemia-reperfusion injury, mainly by lowering the number of circulating neutrophils [73] or autophagy induction, which may decrease the secretion of proinflammatory cytokines [74]. Indeed, Eleftheriadis et al. reported that Trp deprivation increased renal proximal tubular epithelial cell survival in hypoxic conditions through autophagy induction [75]. Further studies are needed to clarify the impact of Trp on kidney graft preservation results. 

## 3. Immune Function in Kidney Transplantation and the Kynurenine Pathway

Despite significant progress in immunosuppressive treatment and methods of immunological monitoring, the 10-year graft survival rate remains suboptimal, reaching 53.6% in patients with kidney transplants received from deceased donors [76]. Chronic antibody-mediated rejection (ABMR) is considered the most common cause of graft loss, without clear benefits from currently available treatment strategies, including intensified immunosuppression or therapeutic plasma exchange (TPE). Therefore, it is of special importance to search for novel targets for the monitoring and treatment of immune-mediated kidney graft damage. 

### 3.1. Indoleamine 2,3-Dioxygenase (IDO)

**Figure 2:**
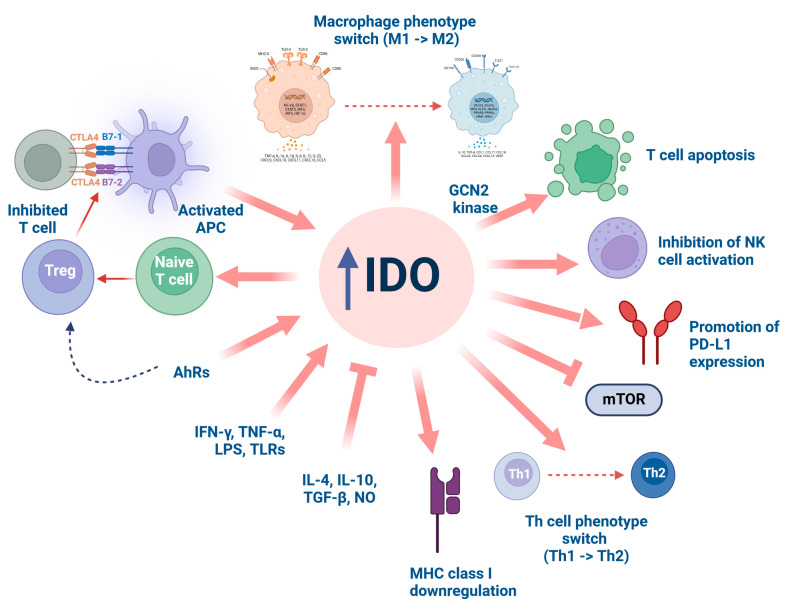
IDO activation resulting in immune cell activity modifications. AhR, aryl hydrocarbon receptor; APC, antigen-presenting cell; CTLA4, cytotoxic T-lymphocyte associated protein 4; GCN2, general control non-derepressible 2; IDO, indoleamine 2,3-dioxygenase; IFN-γ, interferon-γ; IL-4, interleukin-4; IL-10, interleukin-10; LPS, lipopolysaccharide; MHC, major histocompatibility complex; mTOR, mammalian target of rapamycin; NK, natural killer; NO, nitric oxide; PD-L1, programmed death-ligand 1; TGF-β, transforming growth factor-β; Th, T helper cell; TLR, toll-like receptor; TNF-α, tumor necrosis factor-α; Treg, regulatory T cell. Created with https://www.BioRender.com (accessed on 1 August 2024).

IDO remains one of the most extensively studied enzymes in the KYN pathway. The interest in this field has rapidly increased since Munn et al. showed that female mice accepted semi-allogenic fetuses under undisturbed Trp catabolism only [77]. Due to pleiotropic immunoregulatory effects revealed through the years, IDO is considered one of the main guardians of immune tolerance, contributing to the downregulation of allogenic responses (Figure 2). 

IDO is expressed constitutively in the majority of cells and tissues, including immunocompetent cells such as monocytes, macrophages, and DCs. In the kidney, IDO expression was mainly detected in renal tubular epithelial cells [78], which are critically involved in kidney graft rejection, as well as in mesangial [79] or glomerular endothelial cells, as shown in patients with graft glomerulitis [80]. However, IDO localization in the graft may vary through time. Considerable IDO expression was detected around atrophic tubules, in cells resembling endothelial and mesangial cells, and in minor quantities in tubular epithelial cells in biopsy samples obtained from patients at two years after kidney transplantation and with signs of chronic damage [81]. IDO activity is highly susceptible to stimulation with pro-inflammatory stimuli, such as IFN-γ, TNF-α, and TLRs, whereas anti-inflammatory cytokines IL-4 [82], IL-10, transforming growth factor-β (TGF-β) [83], and nitric oxide (NO) [84] diminish it. Among the possible effects of IDO on the immune system, its influence on T cells was widely explored. Stimulation of IDO resulting in Trp depletion was shown to activate the general control non-derepressible 2 (GCN2) kinase pathway, causing an arrest of T cell proliferation in the mid-G1 phase and making them susceptible to apoptosis [85]. Interestingly, apoptosis of T helper (Th1) cells rather than Th2 cells may be evoked by KYN metabolites, 3-HAA and QA [86]. As shown in mesenchymal stem cells, IFN-γ-induced stimulation of IDO results in regulatory T cell (Treg) induction, contributing to local immunosuppressive effects and to attenuation of renal fibrosis [87]. Ligation of B7-1/B7-2 molecules by cytotoxic T-lymphocyte-associated protein 4 (CTLA4)/CD28 present on the surface of Tregs or by a separate CTLA4 fusion protein (CTLA4-Ig) is necessary to activate IDO and afterward inhibit T cell proliferation [88].

IDO activation was also suggested to increase macrophage polarization into the M2 phenotype [89], suppress natural killer (NK) cell activity [90], decrease mean histocompatibility complex (MHC) class I gene expression [91], and be implicated in the development of local immune tolerance. Although IDO does not affect B cell proliferation [92], there is some evidence suggesting the impact of IDO on B cell-driven responses [93]. Inhibition of the mammalian target of rapamycin (mTOR) is another potential mechanism related to IDO’s action [94].

Furthermore, IDO1 may modulate immunological system activity through other mechanisms. The activation of AhR, a transcription factor crucial for immune system control, increases both IDO1 and IDO2 expression in DCs, together with the generation of FoxP3(+) Tregs [95]. On the other hand, it was demonstrated that IDO products alone, especially KYN, can activate AhR and lead to Treg generation [96]. Moreover, in plasmacytoid DCs, TGF-β-dependent self-tolerance was linked with the non-enzymatic regulatory activity of IDO [97]. 

There is ongoing debate about the significance of the KYN pathway activity measurement in kidney transplant recipients. The key question remains whether elevated Trp degradation and production of KYN-derived compounds is an immunosuppressive mechanism preventing tissue damage or rather reflecting already ongoing tissue breakdown. Discrepancies come from different models tested (animal vs. human), diverse tissues and body fluids analyzed (serum, urine, or graft biopsy samples), or analytical methods. Another issue that should be taken into consideration is the kidney transplant recipient’s condition. Lower serum Trp, which is considered a marker of IDO activation, may not reflect true amino acid turnover. After kidney transplantation, based on improved protein ingestion and kidney function normalization, an elevation of serum Trp level should be expected [98]. Indeed, it has been shown that already during the first 3 days after transplantation, changes in Trp and its metabolites can be observed, with a significant reduction of serum KYNA and KYN and an increase of Trp [99]. On the other hand, higher serum Trp can be expected in the case of tissue damage, especially under inflammatory stimuli [100]. The role of gut microbiota in protein breakdown and KYN metabolite formation should not be forgotten. Therefore, the diagnostic and prognostic relevance of the KYN pathway monitoring in kidney transplant recipients remains a huge challenge.

In one of the first studies in kidney transplant recipients, Holmes et al. reported that the serum KYN level started to increase 5 to 7 days prior to a biopsy-confirmed acute rejection (AR) episode, and it did not correlate with serum creatinine concentration or high dose steroid treatment [101]. Later on, it was shown that serum levels of KYN and Trp are higher in non-rejecting allograft recipients than in healthy volunteers, and that their levels considerably increase during rejection episodes [102]. Importantly, the serum KYN/Trp ratio in the rejection group was higher starting from day 1 after transplantation and correlated with serum neopterin concentration, a marker of immune system activation [103]. Moreover, kidney biopsy specimen staining indicated IDO upregulation in tubular epithelial cells of rejected grafts, whereas in healthy grafts, IDO expression was not detectable. Similarly, it was reported that the serum KYN level increases from the 3rd day before biopsy-proven kidney graft rejection, as compared to recipients without rejection episodes [104]. It remains unclear whether the KYN pathway activation can be related to a specific type of graft rejection. In a recently published study, a higher IDO1 expression in interstitial foci of kidney graft biopsies specimens was related to a higher incidence of ABMR (15% vs. 2.44%, *p* = 0.035) and T cell-mediated rejection (TCR) (53.75% vs. 17.07%, *p* = 0.0001) [105]. The significance of IDO elevation in kidney transplant recipients exposed to a plethora of immune stimuli is not fully clarified [106]. Studies in pediatric kidney graft recipients suggested that serum KYN/Trp ratio elevation can be an important tool in differentiating acute graft rejection from infection [107,108]. 

### 3.2. Kynurenine 3-Monooxygenase (KMO)

Possible involvement of KMO in graft survival was recently highlighted. In contrast to IDO, KMO is a mitochondrial outer membrane protein, found preferentially in tubular epithelial cells [109], glomerular cells, and podocytes [110]. KMO is an important enzyme of the KYN pathway, which controls the amount of KYN entering three different arms of the path and thus regulates the formation of various biologically active products, including NAD^+^ (Figure 1). Diminished de novo NAD^+^ formation due to KMO downregulation in proximal tubular epithelial cells was postulated as one of the potential mechanisms of acute kidney injury (AKI) [111]. Direct KMO products, 3-HK together with 3-HAA, are well-known immunosuppressive agents. Both compounds significantly inhibit T cell proliferation and survival without effect on DCs or endothelial cells, as shown in the murine corneal allograft model [112]. Inhibition of KMO, resulting in lower 3-HK and 3-HAA formation, can be related to a higher immunological risk of graft rejection. Indeed, in a porcine model of acute kidney allograft rejection, lower KMO gene transcription and protein level, with concomitant higher IDO gene expression and enzyme activity, were demonstrated [113]. Similarly, a significant reduction in KMO activity was observed in a pig model of kidney allograft rejection [109]. Additionally, 3-HK and 3-HAA protected tubular epithelial cells from injury, in part through inhibition of T cell proliferation and upregulation of AhR expression. Moreover, 3-HK was reported to inhibit mesangial cell proliferation [114], whereas 3-HAA and 3-HK attenuated glomerular injury in an experimental autoimmune glomerulonephritis animal model [115]. 

On the other hand, in animals with the KMO knockout, a proteinuria phenotype developed, and in diabetic patients, a significant decrease in podocytic KMO expression was found [110]. Thus, more studies are needed to explore the role of KMO and its metabolites in kidney diseases.

### 3.3. Kynurenic Acid (KYNA)

The immunomodulatory role of KYNA is well recognized. Through the impact on GPR35, KYNA may inhibit the activation of the NLRP3 inflammasome in macrophages and suppress the production of caspase-1 and IL-1 β [116]. KYNA, through an activation of AhR, was shown to enhance the expression of TNF-stimulated gene 6 (TSG-6) in human mesenchymal stem cells and to reduce immune cell infiltration in an animal model of acute lung injury [58]. The potential impact of KYNA on T cell activity should also be considered, since AhR activation promotes differentiation of Tregs and inhibits expression of proinflammatory IL-17 [117]. Indeed, KYNA was reported to decrease IL-23 secretion by LPS-activated DCs and Th17 cell polarization [118]. Additionally, KYNA lowered human invariant NK T cell activation through GPR35 [119]. However, in an animal model of arthritis, KYNA failed to promote Treg development, contrary to the other NMDA antagonist memantine [120]. Other effects, including inhibition of TNF-α secretion from human mononuclear cells and high mobility group box protein 1 (HMGB1) secretion in monocytes and granulocyte cultures, were shown [121]. Although KYNA was not examined in kidney transplant patients in the context of immunological risk, presented data suggest the involvement of KYNA in immune tolerance formation. Interestingly, it was reported that tacrolimus (TAC), but not cyclosporine (CsA), elevates KYNA formation, suggesting KYNA’s role in selected immunosuppressive drug action [122]. 

## 4. Infections 

In addition to the great impact on alloantigen tolerance induction, the activation of the KYN pathway may impair the immune response against pathogens, which is of special importance in kidney transplant recipients. Trp degradation is a well-described mechanism of suppressing pathogens’ growth, including *Chlamydia* species [123] or cytomegalovirus (CMV) [124]. However, an uncontrolled KYN pathway activation, especially during sepsis, is considered to act bidirectionally. The pro-inflammatory state results in an increased level of certain Trp metabolites and leads to an impaired response to opportunistic infections [125], which may have a tremendous impact on patient outcomes. Indeed, plasma KYNA levels in patients with septic shock correlated with lactate and procalcitonin concentration only in survivors. Furthermore, a lack of reduction of KYNA after continuous veno-venous hemofiltration (CVVH) procedures was suggested as a factor predicting the fatal outcome of septic shock [126]. Although data from kidney transplant recipients are limited, KYN pathway activity analysis was indicated as a promising tool in early diagnosis of infectious complications and evaluation of their intensity. In a group of 355 kidney graft recipients, blood KYN levels were positively correlated with CMV infection severity prior to the diagnosis and were higher in patients with bacterial sepsis [106]. A decrease in blood KYN concentration reflected a response to the treatment, whereas its further increase was associated with poor outcomes. Similarly, serum KYN levels were markedly elevated in kidney transplant recipients with viral and Gram-negative bacterial infections in the absence of AR, making this parameter useful in differentiating posttransplant complications [101]. Interestingly, KYN and QA plasma concentrations were shown to increase significantly during CMV, but not BKV infection, indicating their significance in the fast detection of systemic immune activation linked with CMV [127]. More studies are needed to explore the relevance of KYN pathway metabolite measurements in different types of infections after kidney transplantation and its impact on long-term outcomes. 

## 5. Malignancies

Despite the well-known effects of KYN pathway activation in immune tolerance, Trp degradation can be a double-edged sword regarding carcinogenesis, especially in kidney transplant recipients. In the tumor microenvironment, a complex interplay between immune and cancer cells regulates tumor growth, its response to treatment, and metastasis formation. In clear cell renal cell carcinoma (RCC), secreted IL-10 and TGF-β promote Treg differentiation, which suppresses effector T cells and stimulates tumor growth [128]. Increased PD-L1 (programmed death-ligand 1) expression is another mechanism of negative T cell function control by cancer cells [129]. It was demonstrated that PD-L1 expression is enhanced by IDO1 in cancer cells in an AhR-dependent manner [130] and reduced by IDO1/TDO inhibition [131]. Co-expression of PD-L1 and IDO1 are negative prognostic factors associated with poor overall survival [132]. IDO1 and IDO2 were found in various types of cancers [133], and their presence was related to resistance to therapy and lower survival. A higher KYN/Trp ratio in clear cell RCC patients was associated with a decreased 5-year survival compared to subjects with low KYN/Trp values [134]. Other abnormalities in KYN pathway activity in tissues of RCC patients include lower expression of QPRT and downregulation of KMO and 3-HAAO [135], although QPRT activity may vary in different types of cancers [136]. Under QPRT inhibition, NAD^+^ synthesis is secured by the activation of a salvage pathway, often preferred by cancer cells, in which nicotinamide is converted by nicotinamide phosphoribosyltransferase (NAMPT) into NAD^+^ [137]. In patients with metastatic RCC, increased tissue TDO expression was strongly associated with higher KYN levels, a worse response to immunotherapy, and higher mortality [138]. Therefore, the KYN pathway can be an interesting target for diagnosis, monitoring, and treatment of malignancies [139]. In fact, IDO [140], TDO [141], or KMO [142] inhibitors were found effective as antineoplastic agents in certain types of cancer [143]. However, inhibitors of the KYN pathway enzymes should be used with great caution in kidney transplant recipients due to the potential risk of AR, as observed with programmed cell death protein 1 (PD-1), PDL-1, and CTLA-4 inhibitors [144]. On the other hand, KYNA exhibited antiproliferative effects on glioblastoma [145], colon [146], and renal [147] cancer cells, but its potential role in carcinogenesis is not fully clarified [148]. Therefore, a more selective KYN pathway inhibition should be preferred. 

## 6. Cardiovascular Risk

KYN pathway metabolites play a complex role in cardiovascular disease pathogenesis. Although Trp degradation products possess anti- and pro-inflammatory properties, most studies correlated them with higher cardiovascular risk, especially due to AhR activation [23]. Recent animal studies suggested a direct effect of KYN on blood pressure elevation and kidney damage [149]. Elevated Trp breakdown was associated with the development of cardiovascular disease [22] and was suggested as a prognostic factor in heart failure [150], coronary artery disease [151], pulmonary hypertension [152], and thromboembolic episodes [153], especially in patients with established CKD. In a recent Chronic Kidney Disease–Renal Epidemiology and Information Network (CKD-REIN) study, KYN was established as an independent risk factor for nonfatal and fatal cardiovascular outcomes in patients with CKD [154]. In this cohort of patients, increased Trp catabolism was correlated with procoagulant factors including plasma tissue factor [155], prothrombin fragments 1 + 2 [156], thrombomodulin, and von Willebrand factor level [157]. Although more evidence indicates the involvement of KYN metabolites in tissue damage, KYNA displays distinct properties. In bovine endothelial cell cultures, KYNA prevented cells from homocysteine-induced impairment, significantly increasing their migration and proliferation [158]. A positive correlation between KYNA plasma concentration and hyperhomocysteinemia in peritoneal dialyzed patients with atherosclerotic cardiovascular disease, possibly reflecting the protective effects of KYNA in this group of patients, was reported [159]. A relationship between serum KYNA level and aortic stiffness in patients with atrial fibrillation was also presented [160].

In kidney transplant recipients, cardiovascular diseases remain the most common cause of death, accounting for about 20–35% of overall mortality and significantly decreasing life expectancy [161]. Therefore, the search for novel diagnostic and therapeutic targets in this population is of special importance. Due to the fact that serum KYN and 3-HK levels were shown to be independently associated with graft failure, and 3-HK level was linked with higher mortality in kidney transplant recipients [100], more studies are necessary to explore the impact of the KYN pathway activation on cardiovascular risk and mortality in this particular group of patients. 

A summary of possible effects of KYN pathway activity alterations in kidney transplant recipients is presented in Table 1. 

## 7. Pharmacological Interventions

**Figure 3:**
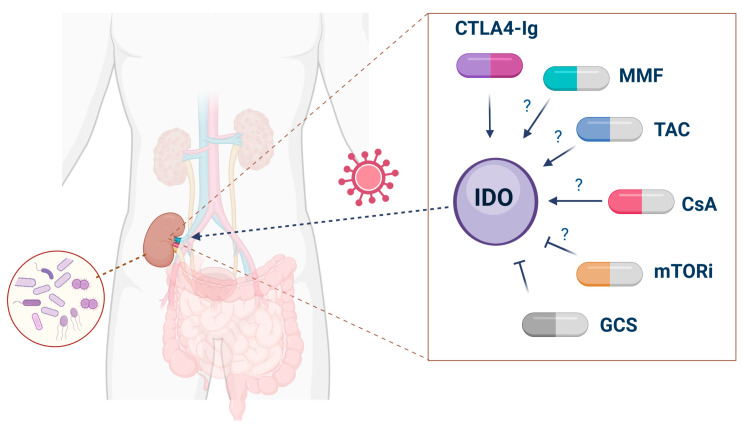
Modulation of the KYN pathway activity in kidney transplant recipients. The figure presents possible effects of immunosuppressive treatment on IDO activity, as well as other methods of KYN pathway modifications, through the impact on urinary microbiota (**left**) or local IDO transfection (**right**). CsA, cyclosporine A; CTLA4-lg, cytotoxic T-lymphocyte associated protein 4 fusion protein; GCS, glucocorticosteroids; IDO, indoleamine-2,3-dioxygenase; MMF, mycophenolate mofetil; mTORi, mammalian target of rapamycin inhibitor; TAC, tacrolimus. Created with https://www.BioRender.com (accessed on 1 August 2024).

The impact of pharmacotherapy, in particular immunosuppression, on the KYN pathway activity after kidney transplantation is a matter of debate (Figure 3).

Since increased Trp metabolism along the KYN pathway induces immune tolerance, the impact of immunosuppressive drugs on the pathway seems conceivable. On the other hand, immunosuppressive agents can potentially restrict IDO overactivation. Changes in the KYN pathway activity may also serve as a diagnostic tool assessing the response to the pharmacotherapy, similarly to selected soluble checkpoint inhibitors in patients with antineutrophil cytoplasmic antibody (ANCA)-associated vasculitis (AAV) [162]. 

So far, the data on immunomodulating agents and the KYN pathway in kidney transplantation are rather limited. In phytohemagglutinin-stimulated and unstimulated human peripheral blood mononuclear cells, TAC, CsA, mammalian target of rapamycin inhibitor (mTORi) sirolimus, and methylprednisolone dose-dependently inhibited Trp degradation and neopterin level, whereas mycophenolate mofetil (MMF) was efficacious only at higher concentrations [163]. Different results were obtained in a mesangial cell line, in which incubation with MMF, CsA, and TAC significantly enhanced IDO expression, but sirolimus showed no effect [79]. As previously mentioned, TAC was shown to increase KYNA synthesis in cortical slices, which suggests that this KYN metabolite may be involved in TAC’s action [122]. Prior studies suggested that immunosuppressive drugs may affect the KYN pathway differently in various types of cells and that the outcome depends on coexisting immune stimuli. Studies analyzing the impact of CTLA4-Ig, abatacept and belatacept, on the KYN pathway did not yield consistent results. Abatacept failed to change the expression of IDO mRNA and KYN levels in DCs [164], and CTLA4-Ig did not increase the KYN/Trp ratio in bone marrow recipients [165]. In contrast, recombinant soluble CTLA4 induced the expression of IDO in human peripheral blood mononuclear cells and DCs [166]. Transduction of adenovirus-mediated CTLA4Ig (AdCTLA4Ig) increased IDO mRNA and KYN levels, and both effects were blocked by the IDO inhibitor 1-methylTrp (1-MT) [167]. In kidney graft biopsies specimens, belatacept treatment increased the number of Tregs in dysfunctional kidneys compared to CsA, whereas no changes in peripheral Tregs were observed [168]. Similarly, higher Treg levels, lower CD4(+)/IL-17A cell levels, decreased kidney graft fibrosis [169], and higher peripheral Bregs, Tregs, and DCs counts [170] were demonstrated after belatacept treatment in comparison to patients receiving CsA. Although an important alternative to calcineurin inhibitors, beletacept use is still limited due to the increased risk of AR and posttransplant lymphoproliferative disorder [171].

On the other hand, targeted KYN pathway inhibition may provide beneficial effects on the treatment of selected complications after kidney transplantation, namely malignancies or cardiovascular diseases. Beyond KMO, KATs, and KYNU inhibitors, which are potentially novel candidates in anticancer therapy [136], other already available drugs were shown to selectively inhibit the KYN pathway. Angiotensin converting enzyme inhibitors [172], angiotensin II type 1 receptor blockers [173], cyclooxygenase inhibitors [174], fibrates [175], and glibenclamide [176] were reported to inhibit KATs activity, which may result in the reduction of kidney damage-related complications, especially in kidney transplant recipients.

## 8. Conclusions

Generalized KYN pathway activation with the goal to evoke peripheral immune tolerance poses a risk of uncontrolled immune system depression; therefore, methods of its localized activation are of special interest. Local administration of IDO by viral vectors [177,178] or modulation of urinary microbiota [179] are interesting options for future studies on the modulation of KYN pathway activity in kidney transplant recipients. 

Exploring KYN pathway activity in patients after kidney transplantation offers an interesting approach to the diagnosis and treatment of tissue graft rejection and related complications. Due to the multiple and often opposing effects of the KYN pathway metabolites on cellular survival and immune processes, there is a need for enzyme- and target-organ-selective pharmacological tools. Precise targeting of the pathway may bring novel diagnostic and therapeutic tools, allowing better management of kidney transplant recipients. 

## Figures and Tables

**Table 1:** Summary of KYN pathway activity alterations and their possible effects on kidney transplant recipients. ABMR, antibody-mediated rejection; CKD, chronic kidney disease; IDO, indoleamine-2,3,-dioxygenase; KMO, kynurenine 3-monooxygenase; KYN, kynurenine; KYNA, kynurenic acid; RCC, renal cell carcinoma; Tac, tacrolimus; TCR, T-cell-mediated rejection; Trp, tryptophan.

KYN Pathway Element	Organ or System Function Modification
Indoleamine-2,3 dioxygenase (IDO)	High IDO activity correlates with chronic kidney disease (CKD) severity and inflammatory parameters [63]; IDO activity measurement as a tool for early CKD diagnosis [69]; IDO upregulation in tubular epithelial cells of rejected kidney grafts [102]; Higher IDO expression in kidney graft biopsies related to a higher incidence of antibody-mediated rejection (ABMR) or T-cell-mediated rejection (TCR) [105]; Elevated kynurenine (KYN)/tryptophan (Trp) ratio as a tool in differentiating acute graft rejection from infection [107,108]; High IDO expression in various cancers related to resistance to anticancer treatment and lower survival [133]; High IDO activity related to cardiovascular risk [23].
Kynurenine 3-monooxygenase (KMO)	Low KMO gene expression protein level [113] and activity [109] in a porcine model of kidney allograft rejection; KMO knockout results in proteinuria in animal models [110]; Low podocytic KMO expression in diabetic patients [110]; Low KMO expression in renal cell carcinoma (RCC) patients [135]; KMO inhibitors as antineoplastic drugs in certain types of cancer [142].
Kynurenic acid (KYNA)	High serum KYNA level correlates with CKD severity and inflammatory markers [63]; High plasma KYNA levels correlate with procalcitonin and lactate concentration in survivors from septic shock, whereas a lack of KYNA concentration reduction relates to poor survival [126]; Plasma KYNA concentration correlates with hyperhomocysteinemia in peritoneal dialyzed patients with atherosclerotic cardiovascular disease [159]; Serum KYNA level related to aortic stiffness in patients with atrial fibrillation [160]; Tacrolimus Tac increases KYNA production [122].

## Abbreviations

1-MT	1-methyltryptophan
3-HAA	3-hydroxyanthranilic acid
3-HAAO	3-hydroxyanthranilic acid dioxygenase
3-HK	3-hydroxykynurenine
AA	anthranilic acid
AAV	ANCA-associated vasculitis
ABMR	antibody-mediated rejection
AdCTLA4Ig	adenovirus-mediated CTLA4Ig
AhR	aryl hydrocarbon receptor
AKI	acute kidney injury
AMPA	alpha-amino-3-hydroxy-5-methyl-4-isoxazolepropionic acid
ANCA	antineutrophil cytoplasmic antibody
AR	acute rejection
BKV	polyomavirus BK
CKD	chronic kidney disease
CTLA4	cytotoxic T-lymphocyte-associated protein 4
CTLA4-Ig	CTLA4 fusion protein
CMV	cytomegalovirus
CsA	cyclosporine
CVVH	continuous veno-venous hemofiltration
DC	dendritic cell
dd-cfDNA	donor-derived cell-free DNA
ESKD	end stage kidney disease
GCN2	general control non-derepressible 2
GCS	glucocorticosteroids
GLU	glutamate
GPR35	G protein-coupled receptor 35
HIV	human immunodeficiency virus
HMGB1	high mobility group box protein 1
hsCRP	high-sensitivity C-reactive protein
HTK	histidine-tryptophan-ketoglutarate
IDO	indoleamine-2,3-dioxygenase
IDO1	indoleamine-2,3-dioxygenase 1
IDO2	indoleamine-2,3-dioxygenase 2
IFN-γ	interferon-γ
IL	interleukin
KAT	kynurenine aminotransferase
KMO	kynurenine 3-monooxygenase
KRT	kidney replacement therapy
KYN	kynurenine
KYNA	kynurenic acid
KYNU	kynureninase
LPS	lipopolysaccharide
MHC	mean histocompatibility complex
MMF	mycophenolate mofetil
mTOR	mammalian target of rapamycin
mTORi	mammalian target of rapamycin inhibitor
NAD^+^	nicotinamide adenine dinucleotide
NAMPT	nicotinamide phosphoribosyltransferase
N-formyl-KYN	N-formyl-kynurenine
NK	natural killer
NMDA	N-methyl-D-aspartate
NO	nitric oxide
PA	picolinic acid
PD-1	programmed cell death protein 1
PD-L1	programmed death-ligand 1
QA	quinolinic acid
QPRT	quinolinic acid phosphoribosyltransferase
RCC	renal cell carcinoma
sTNF-1	soluble TNF-receptor-1
TAC	tacrolimus
TCR	T cell mediated rejection
TDO	tryptophan-2,3-dioxygenase
TGF-β	transforming growth factor-β
Th	T helper cell
TLR	toll like receptor
TNF-α	tumor necrosis factor-α
TPE	therapeutic plasma exchange
Treg	regulatory T cell
Trp	tryptophan
TSG-6	TNF-stimulated gene 6
XA	xanthurenic acid

## References

[1] Jager K.J., Kovesdy C., Langham R., Rosenberg M., Jha V., Zoccali C. (2019). A Single Number for Advocacy and Communication-Worldwide More than 850 Million Individuals Have Kidney Diseases. Nephrol. Dial. Transpl..

[2] Foreman K.J., Marquez N., Dolgert A., Fukutaki K., Fullman N., McGaughey M., Pletcher M.A., Smith A.E., Tang K., Yuan C.-W. (2018). Forecasting Life Expectancy, Years of Life Lost, and All-Cause and Cause-Specific Mortality for 250 Causes of Death: Reference and Alternative Scenarios for 2016-40 for 195 Countries and Territories. Lancet.

[3] Astley M., Caskey F.J., Evans M., Torino C., Szymczak M., Drechsler C., Pippias M., de Rooij E., Porto G., Stel V.S. (2023). The Impact of Gender on the Risk of Cardiovascular Events in Older Adults with Advanced Chronic Kidney Disease. Clin. Kidney J..

[4] Kim J.Y., Steingroever J., Lee K.H., Oh J., Choi M.J., Lee J., Larkins N.G., Schaefer F., Hong S.H., Jeong G.H. (2020). Clinical Interventions and All-Cause Mortality of Patients with Chronic Kidney Disease: An Umbrella Systematic Review of Meta-Analyses. J. Clin. Med..

[5] Thurlow J.S., Joshi M., Yan G., Norris K.C., Agodoa L.Y., Yuan C.M., Nee R. (2021). Global Epidemiology of End-Stage Kidney Disease and Disparities in Kidney Replacement Therapy. Am. J. Nephrol..

[6] Stel V.S., Jager K.J., Ortiz A. (2024). Sixty Years of European Renal Association (ERA) Registry Data on Kidney Disease: Visualizing Differences in Clinical Practice. Nephrol. Dial. Transpl..

[7] Boerstra B.A., Boenink R., Astley M.E., Bonthuis M., Abd ElHafeez S., Arribas Monzón F., Åsberg A., Beckerman P., Bell S., Cases Amenós A. (2024). The ERA Registry Annual Report 2021: A Summary. Clin. Kidney J..

[8] Stel V.S., Boenink R., Astley M.E., Boerstra B.A., Radunovic D., Skrunes R., Ruiz San Millán J.C., Slon Roblero M.F., Bell S., Ucio Mingo P. (2024). A Comparison of the Epidemiology of Kidney Replacement Therapy between Europe and the United States: 2021 Data of the ERA Registry and the USRDS. Nephrol. Dial. Transpl..

[9] Cohen-Bucay A., Gordon C.E., Francis J.M. (2019). Non-Immunological Complications Following Kidney Transplantation. F1000Research.

[10] Serkies K., Dębska-Ślizień A., Kowalczyk A., Lizakowski S., Małyszko J. (2023). Malignancies in Adult Kidney Transplant Candidates and Recipients: Current Status. Nephrol. Dial. Transpl..

[11] Zoccali C., Tripepi G., Stel V., Fu E.L., Mallamaci F., Dekker F., Jager K.J. (2024). Biomarkers in Clinical Epidemiology Studies. Clin. Kidney J..

[12] Gupta G., Athreya A., Kataria A. (2024). Biomarkers in Kidney Transplantation: A Rapidly Evolving Landscape. Transplantation.

[13] Park S., Sellares J., Tinel C., Anglicheau D., Bestard O., Friedewald J.J. (2023). European Society of Organ Transplantation Consensus Statement on Testing for Non-Invasive Diagnosis of Kidney Allograft Rejection. Transpl. Int..

[14] Zulpaite R., Miknevicius P., Leber B., Strupas K., Stiegler P., Schemmer P. (2021). Tryptophan Metabolism via Kynurenine Pathway: Role in Solid Organ Transplantation. Int. J. Mol. Sci..

[15] Le Floc’h N., Otten W., Merlot E. (2011). Tryptophan Metabolism, from Nutrition to Potential Therapeutic Applications. Amino Acids.

[16] Németh H., Toldi J., Vécsei L. (2005). Role of Kynurenines in the Central and Peripheral Nervous Systems. Curr. Neurovasc. Res..

[17] Mor A., Tankiewicz-Kwedlo A., Krupa A., Pawlak D. (2021). Role of Kynurenine Pathway in Oxidative Stress during Neurodegenerative Disorders. Cells.

[18] Lashgari N.-A., Roudsari N.M., Shayan M., Niazi Shahraki F., Hosseini Y., Momtaz S., Abdolghaffari A.H. (2023). IDO/Kynurenine; Novel Insight for Treatment of Inflammatory Diseases. Cytokine.

[19] Mor A., Tankiewicz-Kwedlo A., Pawlak D. (2021). Kynurenines as a Novel Target for the Treatment of Malignancies. Pharmaceuticals.

[20] Zakrocka I., Załuska W. (2022). Kynurenine Pathway in Kidney Diseases. Pharmacol. Rep..

[21] Mor A., Kalaska B., Pawlak D. (2020). Kynurenine Pathway in Chronic Kidney Disease: What’s Old, What’s New, and What’s Next?. Int. J. Tryptophan Res..

[22] Teunis C.J., Stroes E.S.G., Boekholdt S.M., Wareham N.J., Murphy A.J., Nieuwdorp M., Hazen S.L., Hanssen N.M.J. (2023). Tryptophan Metabolites and Incident Cardiovascular Disease: The EPIC-Norfolk Prospective Population Study. Atherosclerosis.

[23] Yang Y., Liu X., Liu X., Xie C., Shi J. (2024). The Role of the Kynurenine Pathway in Cardiovascular Disease. Front. Cardiovasc. Med..

[24] Kiluk M., Lewkowicz J., Kowalska I., Pawlak D., Łagoda K., Tankiewicz-Kwedlo A. (2023). Alterations of the Kynurenine Pathway in Patients with Type 1 Diabetes are Associated with Metabolic Control of Diabetes. Pol. Arch. Intern. Med..

[25] Kozieł K., Urbanska E.M. (2023). Kynurenine Pathway in Diabetes Mellitus—Novel Pharmacological Target?. Cells.

[26] Hainz U., Jürgens B., Heitger A. (2007). The Role of Indoleamine 2,3-Dioxygenase in Transplantation. Transpl. Int..

[27] Ren S., Correia M.A. (2000). Heme: A Regulator of Rat Hepatic Tryptophan 2,3-Dioxygenase?. Arch. Biochem. Biophys..

[28] Rodriguez Cetina Biefer H., Vasudevan A., Elkhal A. (2017). Aspects of Tryptophan and Nicotinamide Adenine Dinucleotide in Immunity: A New Twist in an Old Tale. Int. J. Tryptophan Res..

[29] Knox W.E., Mehler A.H. (1951). The Adaptive Increase of the Tryptophan Peroxidase-Oxidase System of Liver. Science.

[30] Knox W.E., Auerbach V.H. (1955). The Hormonal Control of Tryptophan Peroxidase in the Rat. J. Biol. Chem..

[31] Schutz G., Feigelson P. (1972). Purification and Properties of Rat Liver Tryptophan Oxygenase. J. Biol. Chem..

[32] Carlin J.M., Borden E.C., Sondel P.M., Byrne G.I. (1989). Interferon-Induced Indoleamine 2,3-Dioxygenase Activity in Human Mononuclear Phagocytes. J. Leukoc. Biol..

[33] Hwu P., Du M.X., Lapointe R., Do M., Taylor M.W., Young H.A. (2000). Indoleamine 2,3-Dioxygenase Production by Human Dendritic Cells Results in the Inhibition of T Cell Proliferation. J. Immunol..

[34] Yoshida R., Imanishi J., Oku T., Kishida T., Hayaishi O. (1981). Induction of Pulmonary Indoleamine 2,3-Dioxygenase by Interferon. Proc. Natl. Acad. Sci. USA.

[35] Babcock T.A., Carlin J.M. (2000). Transcriptional Activation of Indoleamine Dioxygenase by Interleukin 1 and Tumor Necrosis Factor Alpha in Interferon-Treated Epithelial Cells. Cytokine.

[36] Fallarino F., Pallotta M.T., Matino D., Gargaro M., Orabona C., Vacca C., Mondanelli G., Allegrucci M., Boon L., Romani R. (2015). LPS-Conditioned Dendritic Cells Confer Endotoxin Tolerance Contingent on Tryptophan Catabolism. Immunobiology.

[37] Thomas S.R., Stocker R. (1999). Redox Reactions Related to Indoleamine 2,3-Dioxygenase and Tryptophan Metabolism along the Kynurenine Pathway. Redox Rep..

[38] Takikawa O., Yoshida R., Kido R., Hayaishi O. (1986). Tryptophan Degradation in Mice Initiated by Indoleamine 2,3-Dioxygenase. J. Biol. Chem..

[39] Badawy A.A.-B. (2017). Tryptophan Availability for Kynurenine Pathway Metabolism across the Life Span: Control Mechanisms and Focus on Aging, Exercise, Diet and Nutritional Supplements. Neuropharmacology.

[40] Höglund E., Øverli Ø., Winberg S. (2019). Tryptophan Metabolic Pathways and Brain Serotonergic Activity: A Comparative Review. Front. Endocrinol..

[41] Metz R., Duhadaway J.B., Kamasani U., Laury-Kleintop L., Muller A.J., Prendergast G.C. (2007). Novel Tryptophan Catabolic Enzyme IDO2 is the Preferred Biochemical Target of the Antitumor Indoleamine 2,3-Dioxygenase Inhibitory Compound D-1-Methyl-Tryptophan. Cancer Res..

[42] Ball H.J., Yuasa H.J., Austin C.J.D., Weiser S., Hunt N.H. (2009). Indoleamine 2,3-Dioxygenase-2; a New Enzyme in the Kynurenine Pathway. Int. J. Biochem. Cell Biol..

[43] Davis I., Liu A. (2015). What is the Tryptophan Kynurenine Pathway and Why is It Important to Neurotherapeutics?. Expert Rev. Neurother..

[44] Christen S., Peterhans E., Stocker R. (1990). Antioxidant Activities of Some Tryptophan Metabolites: Possible Implication for Inflammatory Diseases. Proc. Natl. Acad. Sci. USA.

[45] Okuda S., Nishiyama N., Saito H., Katsuki H. (1998). 3-Hydroxykynurenine, an Endogenous Oxidative Stress Generator, Causes Neuronal Cell Death with Apoptotic Features and Region Selectivity. J. Neurochem..

[46] Goldstein L.E., Leopold M.C., Huang X., Atwood C.S., Saunders A.J., Hartshorn M., Lim J.T., Faget K.Y., Muffat J.A., Scarpa R.C. (2000). 3-Hydroxykynurenine and 3-Hydroxyanthranilic Acid Generate Hydrogen Peroxide and Promote Alpha-Crystallin Cross-Linking by Metal Ion Reduction. Biochemistry.

[47] Ferreira F.S., Junior O.V.R., Dos Santos T.M., Silveira J.S., Deniz B.F., Alves V.S., Coutinho-Silva R., Savio L.E.B., Wyse A.T.S. (2023). Effect of Quinolinic Acid on Behavior, Morphology, and Expression of Inflammatory/Oxidative Status in Rats’ Striatum: Is Coenzyme Q10 a Good Protector?. Neurotox. Res..

[48] Okuda S., Nishiyama N., Saito H., Katsuki H. (1996). Hydrogen Peroxide-Mediated Neuronal Cell Death Induced by an Endogenous Neurotoxin, 3-Hydroxykynurenine. Proc. Natl. Acad. Sci. USA.

[49] Ganong A.H., Cotman C.W. (1986). Kynurenic Acid and Quinolinic Acid Act at N-Methyl-D-Aspartate Receptors in the Rat Hippocampus. J. Pharmacol. Exp. Ther..

[50] Birch P.J., Grossman C.J., Hayes A.G. (1988). Kynurenate and FG9041 Have Both Competitive and Non-Competitive Antagonist Actions at Excitatory Amino Acid Receptors. Eur. J. Pharmacol..

[51] Ostapiuk A., Urbanska E.M. (2022). Kynurenic Acid in Neurodegenerative Disorders-Unique Neuroprotection or Double-Edged Sword?. CNS Neurosci. Ther..

[52] Alberts C., Owe-Larsson M., Urbanska E.M. (2023). New Perspective on Anorexia Nervosa: Tryptophan-Kynurenine Pathway Hypothesis. Nutrients.

[53] Vamos E., Pardutz A., Klivenyi P., Toldi J., Vecsei L. (2009). The Role of Kynurenines in Disorders of the Central Nervous System: Possibilities for Neuroprotection. J. Neurol. Sci..

[54] Stone T.W., Williams R.O. (2023). Tryptophan Metabolism as a “reflex” Feature of Neuroimmune Communication: Sensor and Effector Functions for the Indoleamine-2, 3-Dioxygenase Kynurenine Pathway. J. Neurochem..

[55] Albuquerque E.X., Schwarcz R. (2013). Kynurenic Acid as an Antagonist of A7 Nicotinic Acetylcholine Receptors in the Brain: Facts and Challenges. Biochem. Pharmacol..

[56] DiNatale B.C., Murray I.A., Schroeder J.C., Flaveny C.A., Lahoti T.S., Laurenzana E.M., Omiecinski C.J., Perdew G.H. (2010). Kynurenic Acid is a Potent Endogenous Aryl Hydrocarbon Receptor Ligand That Synergistically Induces Interleukin-6 in the Presence of Inflammatory Signaling. Toxicol. Sci..

[57] Wang J., Simonavicius N., Wu X., Swaminath G., Reagan J., Tian H., Ling L. (2006). Kynurenic Acid as a Ligand for Orphan G Protein-Coupled Receptor GPR35. J. Biol. Chem..

[58] Wang G., Cao K., Liu K., Xue Y., Roberts A.I., Li F., Han Y., Rabson A.B., Wang Y., Shi Y. (2018). Kynurenic Acid, an IDO Metabolite, Controls TSG-6-Mediated Immunosuppression of Human Mesenchymal Stem Cells. Cell Death Differ..

[59] Wirthgen E., Hoeflich A., Rebl A., Günther J. (2017). Kynurenic Acid: The Janus-Faced Role of an Immunomodulatory Tryptophan Metabolite and Its Link to Pathological Conditions. Front. Immunol..

[60] Kaya B., Melhem H., Niess J.H. (2021). GPR35 in Intestinal Diseases: From Risk Gene to Function. Front. Immunol..

[61] Iwaniak P., Owe-Larsson M., Urbańska E.M. (2024). Microbiota, Tryptophan and Aryl Hydrocarbon Receptors as the Target Triad in Parkinson’s Disease—A Narrative Review. Int. J. Mol. Sci..

[62] Shaw C., Hess M., Weimer B.C. (2023). Microbial-Derived Tryptophan Metabolites and Their Role in Neurological Disease: Anthranilic Acid and Anthranilic Acid Derivatives. Microorganisms.

[63] Schefold J.C., Zeden J.P., Fotopoulou C., Von Haehling S., Pschowski R., Hasper D., Volk H.D., Schuett C., Reinke P. (2009). Increased Indoleamine 2,3-Dioxygenase (IDO) Activity and Elevated Serum Levels of Tryptophan Catabolites in Patients with Chronic Kidney Disease: A Possible Link between Chronic Inflammation and Uraemic Symptoms. Nephrol. Dial. Transpl..

[64] Pawlak K., Kowalewska A., Pawlak D., Mysliwiec M. (2009). Kynurenine and Its Metabolites—Kynurenic Acid and Anthranilic Acid are Associated with Soluble Endothelial Adhesion Molecules and Oxidative Status in Patients with Chronic Kidney Disease. Am. J. Med. Sci..

[65] Pawlak K., Myśliwiec M., Pawlak D. (2010). Kynurenine Pathway—A New Link between Endothelial Dysfunction and Carotid Atherosclerosis in Chronic Kidney Disease Patients. Adv. Med. Sci..

[66] Karu N., McKercher C., Nichols D.S., Davies N., Shellie R.A., Hilder E.F., Jose M.D. (2016). Tryptophan Metabolism, Its Relation to Inflammation and Stress Markers and Association with Psychological and Cognitive Functioning: Tasmanian Chronic Kidney Disease Pilot Study. BMC Nephrol..

[67] Konje V.C., Rajendiran T.M., Bellovich K., Gadegbeku C.A., Gipson D.S., Afshinnia F., Mathew A.V. (2021). Michigan Kidney Translational Core CPROBE Investigator Group Tryptophan Levels Associate with Incident Cardiovascular Disease in Chronic Kidney Disease. Clin. Kidney J..

[68] Benitez T., VanDerWoude E., Han Y., Byun J., Konje V.C., Gillespie B.W., Saran R., Mathew A.V. (2022). Kynurenine Pathway Metabolites Predict Subclinical Atherosclerotic Disease and New Cardiovascular Events in Chronic Kidney Disease. Clin. Kidney J..

[69] Hong H., Zheng J., Shi H., Zhou S., Chen Y., Li M. (2024). Prediction Model for Early-Stage CKD Using the Naples Prognostic Score and Plasma Indoleamine 2,3-Dioxygenase Activity. J. Inflamm. Res..

[70] Bretschneider H.J. (1964). Survival Time and Recuperative Time of The Heart in Normothermia and Hypothermia. Verh. Dtsch. Ges. Kreislaufforsch..

[71] Lee C.Y., Mangino M.J. (2009). Preservation Methods for Kidney and Liver. Organogenesis.

[72] Mohr A., Brockmann J.G., Becker F. (2020). HTK-N: Modified Histidine-Tryptophan-Ketoglutarate Solution-A Promising New Tool in Solid Organ Preservation. Int. J. Mol. Sci..

[73] Peng W., Robertson L., Gallinetti J., Mejia P., Vose S., Charlip A., Chu T., Mitchell J.R. (2012). Surgical Stress Resistance Induced by Single Amino Acid Deprivation Requires Gcn2 in Mice. Sci. Transl. Med..

[74] Fougeray S., Mami I., Bertho G., Beaune P., Thervet E., Pallet N. (2012). Tryptophan Depletion and the Kinase GCN2 Mediate IFN-γ-Induced Autophagy. J. Immunol..

[75] Eleftheriadis T., Pissas G., Sounidaki M., Antoniadis N., Antoniadi G., Liakopoulos V., Stefanidis I. (2017). Preconditioning of Primary Human Renal Proximal Tubular Epithelial Cells without Tryptophan Increases Survival under Hypoxia by Inducing Autophagy. Int. Urol. Nephrol..

[76] Balakrishnan S., Alexander M.P., Schinstock C. (2024). Challenges and Opportunities for Designing Clinical Trials for Antibody Mediated Rejection. Front. Transpl..

[77] Munn D.H., Zhou M., Attwood J.T., Bondarev I., Conway S.J., Marshall B., Brown C., Mellor A.L. (1998). Prevention of Allogeneic Fetal Rejection by Tryptophan Catabolism. Science.

[78] Mohib K., Guan Q., Diao H., Du C., Jevnikar A.M. (2007). Proapoptotic Activity of Indoleamine 2,3-Dioxygenase Expressed in Renal Tubular Epithelial Cells. Am. J. Physiol. Ren. Physiol..

[79] Liang G.-B., Luo G.-H., Bao D.-S., Chen A.-J., Zhuang Y.-X., Guo Y.-N., Wang X., Wang Y.-L., Chen Z.-P., Lu Y.-P. (2015). Impact of Immunosuppressive Agents on the Expression of Indoleamine 2,3-Dioxygenase, Heme Oxygenase-1 and Interleukin-7 in Mesangial Cells. Mol. Med. Rep..

[80] Hendriks S.H., Heidt S., Krop J., IJsselsteijn M.E., Eggermont J., Kers J., Reinders M.E.J., Koning F., van Kooten C. (2024). IDO+ Endothelial Cells in Glomeruli of Kidney Transplantation Patients with Glomerulitis. Transpl. Direct.

[81] Vavrincova-Yaghi D., Seelen M.A., Kema I.P., Deelman L.E., Van Der Heuvel M.C., Breukelman H., Van Den Eynde B.J., Henning R.H., Van Goor H., Sandovici M. (2015). Early Posttransplant Tryptophan Metabolism Predicts Long-Term Outcome of Human Kidney Transplantation. Transplantation.

[82] Musso T., Gusella G.L., Brooks A., Longo D.L., Varesio L. (1994). Interleukin-4 Inhibits Indoleamine 2,3-Dioxygenase Expression in Human Monocytes. Blood.

[83] MacKenzie C.R., González R.G., Kniep E., Roch S., Däubener W. (1999). Cytokine Mediated Regulation of Interferon-Gamma-Induced IDO Activation. Adv. Exp. Med. Biol..

[84] Thomas S.R., Mohr D., Stocker R. (1994). Nitric Oxide Inhibits Indoleamine 2,3-Dioxygenase Activity in Interferon-Gamma Primed Mononuclear Phagocytes. J. Biol. Chem..

[85] Lee G.K., Park H.J., Macleod M., Chandler P., Munn D.H., Mellor A.L. (2002). Tryptophan Deprivation Sensitizes Activated T Cells to Apoptosis Prior to Cell Division. Immunology.

[86] Fallarino F., Grohmann U., Vacca C., Bianchi R., Orabona C., Spreca A., Fioretti M.C., Puccetti P. (2002). T Cell Apoptosis by Tryptophan Catabolism. Cell Death Differ..

[87] Kurawaki S., Nakashima A., Ishiuchi N., Kanai R., Maeda S., Sasaki K., Masaki T. (2024). Mesenchymal Stem Cells Pretreated with Interferon-Gamma Attenuate Renal Fibrosis by Enhancing Regulatory T Cell Induction. Sci. Rep..

[88] Munn D.H., Sharma M.D., Mellor A.L. (2004). Ligation of B7-1/B7-2 by Human CD4+ T Cells Triggers Indoleamine 2,3-Dioxygenase Activity in Dendritic Cells. J. Immunol..

[89] Wang X.-F., Wang H.-S., Wang H., Zhang F., Wang K.-F., Guo Q., Zhang G., Cai S.-H., Du J. (2014). The Role of Indoleamine 2,3-Dioxygenase (IDO) in Immune Tolerance: Focus on Macrophage Polarization of THP-1 Cells. Cell. Immunol..

[90] Kai S., Goto S., Tahara K., Sasaki A., Kawano K., Kitano S. (2003). Inhibition of Indoleamine 2,3-dioxygenase Suppresses NK Cell Activity and Accelerates Tumor Growth. J. Exp. Ther. Oncol..

[91] Li Y., Tredget E.E., Ghahary A. (2004). Cell Surface Expression of MHC Class I Antigen is Suppressed in Indoleamine 2,3-Dioxygenase Genetically Modified Keratinocytes: Implications in Allogeneic Skin Substitute Engraftment. Hum. Immunol..

[92] Frumento G., Rotondo R., Tonetti M., Damonte G., Benatti U., Ferrara G.B. (2002). Tryptophan-Derived Catabolites are Responsible for Inhibition of T and Natural Killer Cell Proliferation Induced by Indoleamine 2,3-Dioxygenase. J. Exp. Med..

[93] Merlo L.M.F., Peng W., Mandik-Nayak L. (2022). Impact of IDO1 and IDO2 on the B Cell Immune Response. Front. Immunol..

[94] Metz R., Rust S., Duhadaway J.B., Mautino M.R., Munn D.H., Vahanian N.N., Link C.J., Prendergast G.C. (2012). IDO Inhibits a Tryptophan Sufficiency Signal That Stimulates MTOR: A Novel IDO Effector Pathway Targeted by D-1-Methyl-Tryptophan. Oncoimmunology.

[95] Vogel C.F.A., Goth S.R., Dong B., Pessah I.N., Matsumura F. (2008). Aryl Hydrocarbon Receptor Signaling Mediates Expression of Indoleamine 2,3-Dioxygenase. Biochem. Biophys. Res. Commun..

[96] Mezrich J.D., Fechner J.H., Zhang X., Johnson B.P., Burlingham W.J., Bradfield C.A. (2010). An Interaction between Kynurenine and the Aryl Hydrocarbon Receptor Can Generate Regulatory T Cells. J. Immunol..

[97] Pallotta M.T., Orabona C., Volpi C., Vacca C., Belladonna M.L., Bianchi R., Servillo G., Brunacci C., Calvitti M., Bicciato S. (2011). Indoleamine 2,3-Dioxygenase is a Signaling Protein in Long-Term Tolerance by Dendritic Cells. Nat. Immunol..

[98] Myśliwiec P., Myśliwiec H., Pawlak D., Dadan J., Buczko W., Pawlak K. (2009). Tryptophan and Its Metabolites in Renal Allograft Recipients. Przegl. Lek..

[99] Te Linde E., van Roij C.J.M., Meijers B.K.I., De Loor H., Kessels R.P.C., Wetzels J.F.M. (2020). Cognitive Function and Uremic Toxins after Kidney Transplantation: An Exploratory Study. Kidney360.

[100] de Vries L.V., Minović I., Franssen C.F.M., van Faassen M., Sanders J.-S.F., Berger S.P., Navis G., Kema I.P., Bakker S.J.L. (2017). The Tryptophan/Kynurenine Pathway, Systemic Inflammation, and Long-Term Outcome after Kidney Transplantation. Am. J. Physiol. Ren. Physiol..

[101] Holmes E.W., Russell P.M., Kinzler G.J., Reckard C.R., Flanigan R.C., Thompson K.D., Bermes E.W. (1992). Oxidative Tryptophan Metabolism in Renal Allograft Recipients: Increased Kynurenine Synthesis is Associated with Inflammation and OKT3 Therapy. Cytokine.

[102] Brandacher G., Cakar F., Winkler C., Schneeberger S., Obrist P., Bösmüller C., Werner-Felmayer G., Werner E.R., Bonatti H., Margreiter R. (2007). Non-Invasive Monitoring of Kidney Allograft Rejection through IDO Metabolism Evaluation. Kidney Int..

[103] Gieseg S.P., Baxter-Parker G., Lindsay A. (2018). Neopterin, Inflammation, and Oxidative Stress: What Could We Be Missing?. Antioxidants.

[104] Lahdou I., Sadeghi M., Daniel V., Schenk M., Renner F., Weimer R., Löb S., Schmidt J., Mehrabi A., Schnitzler P. (2010). Increased Pretransplantation Plasma Kynurenine Levels Do Not Protect from but Predict Acute Kidney Allograft Rejection. Hum. Immunol..

[105] Wiśnicki K., Donizy P., Kuriata-Kordek M., Uchmanowicz I., Zachciał J., Hałoń A., Janczak D., Banasik M. (2024). Interstitial Foci Expression of Indoleamine 2,3-Dioxygenase 1: A Potential Biomarker for Kidney Transplant Rejection. J. Clin. Med..

[106] Kaden J., Abendroth D., Völp A., Marzinzig M. (2015). Dynamics and Diagnostic Relevance of Kynurenine Serum Level after Kidney Transplantation. Ann. Transpl..

[107] Dharnidharka V.R., Gupta S., Al Khasawneh E., Haafiz A., Shuster J.J., Theriaque D.W., Shahlaee A.H., Garrett T.J. (2011). Immune Biomarker Panel Monitoring Utilizing IDO Enzyme Activity and CD4 ATP Levels: Prediction of Acute Rejection vs. Viral Replication Events. Pediatr. Transpl..

[108] Dharnidharka V.R., Al Khasawneh E., Gupta S., Shuster J.J., Theriaque D.W., Shahlaee A.H., Garrett T.J. (2013). Verification of Association of Elevated Serum Ido Enzyme Activity with Acute Rejection and Low Cd4-Atp Levels with Infection. Transplantation.

[109] Lassiter R., Merchen T.D., Fang X., Wang Y. (2021). Protective Role of Kynurenine 3-Monooxygenase in Allograft Rejection and Tubular Injury in Kidney Transplantation. Front. Immunol..

[110] Korstanje R., Deutsch K., Bolanos-Palmieri P., Hanke N., Schroder P., Staggs L., Bräsen J.H., Roberts I.S.D., Sheehan S., Savage H. (2016). Loss of Kynurenine 3-Mono-Oxygenase Causes Proteinuria. J. Am. Soc. Nephrol..

[111] Zhai Y., Chavez J.A., D’Aquino K.E., Meng R., Nawrocki A.R., Pocai A., Wang L., Ma L.-J. (2024). Kynurenine 3-Monooxygenase Limits de Novo NAD+ Synthesis through Dietary Tryptophan in Renal Proximal Tubule Epithelial Cell Models. Am. J. Physiol. Cell Physiol..

[112] Zaher S.S., Germain C., Fu H., Larkin D.F.P., George A.J.T. (2011). 3-Hydroxykynurenine Suppresses CD4+ T-Cell Proliferation, Induces T-Regulatory-Cell Development, and Prolongs Corneal Allograft Survival. Investig. Ophthalmol. Vis. Sci..

[113] Wang Y., Merchen T.D., Fang X., Lassiter R., Ho C.-S., Jajosky R., Kleven D., Thompson T., Mohamed E., Yu M. (2018). Regulation of Indoleamine 2,3 Dioxygenase and Its Role in a Porcine Model of Acute Kidney Allograft Rejection. J. Investig. Med..

[114] Yoshimura H., Sakai T., Kuwahara Y., Ito M., Tsuritani K., Hirasawa Y., Nagamatsu T. (2009). Effects of Kynurenine Metabolites on Mesangial Cell Proliferation and Gene Expression. Exp. Mol. Pathol..

[115] Hou W., Huang G., Cao X., Zhang Y., Zhang J., Li Y. (2014). Suppression of Experimental Autoimmune Glomerulonephritis by Tryptophan. J. Nephrol..

[116] Sun T., Xie R., He H., Xie Q., Zhao X., Kang G., Cheng C., Yin W., Cong J., Li J. (2022). Kynurenic Acid Ameliorates NLRP3 Inflammasome Activation by Blocking Calcium Mobilization via GPR35. Front. Immunol..

[117] Singh N.P., Singh U.P., Singh B., Price R.L., Nagarkatti M., Nagarkatti P.S. (2011). Activation of Aryl Hydrocarbon Receptor (AhR) Leads to Reciprocal Epigenetic Regulation of FoxP3 and IL-17 Expression and Amelioration of Experimental Colitis. PLoS ONE.

[118] Salimi Elizei S., Poormasjedi-Meibod M.-S., Wang X., Kheirandish M., Ghahary A. (2017). Kynurenic Acid Downregulates IL-17/1L-23 Axis in Vitro. Mol. Cell. Biochem..

[119] Fallarini S., Magliulo L., Paoletti T., de Lalla C., Lombardi G. (2010). Expression of Functional GPR35 in Human INKT Cells. Biochem. Biophys. Res. Commun..

[120] Lindblad S.S., Mydel P., Hellvard A., Jonsson I.-M., Bokarewa M.I. (2012). The N-Methyl-d-Aspartic Acid Receptor Antagonist Memantine Ameliorates and Delays the Development of Arthritis by Enhancing Regulatory T Cells. Neurosignals.

[121] Tiszlavicz Z., Németh B., Fülöp F., Vécsei L., Tápai K., Ocsovszky I., Mándi Y. (2011). Different Inhibitory Effects of Kynurenic Acid and a Novel Kynurenic Acid Analogue on Tumour Necrosis Factor-α (TNF-α) Production by Mononuclear Cells, HMGB1 Production by Monocytes and HNP1-3 Secretion by Neutrophils. Naunyn. Schmiedebergs. Arch. Pharmacol..

[122] Luchowska E., Luchowski P., Wielosz M., Turski W.A., Urbanska E.M. (2003). FK506 Attenuates 1-Methyl-4-Phenylpyridinium- and 3-Nitropropionic Acid-Evoked Inhibition of Kynurenic Acid Synthesis in Rat Cortical Slices. Acta Neurobiol. Exp..

[123] Coles A.M., Reynolds D.J., Harper A., Devitt A., Pearce J.H. (1993). Low-Nutrient Induction of Abnormal Chlamydial Development: A Novel Component of Chlamydial Pathogenesis?. FEMS Microbiol. Lett..

[124] Zhang J., Liu Y., Zhi X., Xu L., Tao J., Cui D., Liu T.F. (2024). Tryptophan Catabolism via the Kynurenine Pathway Regulates Infection and Inflammation: From Mechanisms to Biomarkers and Therapies. Inflamm. Res..

[125] Krupa A., Krupa M.M., Pawlak K. (2022). Kynurenine Pathway-An Underestimated Factor Modulating Innate Immunity in Sepsis-Induced Acute Kidney Injury?. Cells.

[126] Dabrowski W., Kocki T., Pilat J., Parada-Turska J., Malbrain M.L.N.G. (2014). Changes in Plasma Kynurenic Acid Concentration in Septic Shock Patients Undergoing Continuous Veno-Venous Haemofiltration. Inflammation.

[127] Sadeghi M., Lahdou I., Daniel V., Schnitzler P., Fusch G., Schefold J.C., Zeier M., Iancu M., Opelz G., Terness P. (2012). Strong Association of Phenylalanine and Tryptophan Metabolites with Activated Cytomegalovirus Infection in Kidney Transplant Recipients. Hum. Immunol..

[128] Monjaras-Avila C.U., Lorenzo-Leal A.C., Luque-Badillo A.C., D’Costa N., Chavez-Muñoz C., Bach H. (2023). The Tumor Immune Microenvironment in Clear Cell Renal Cell Carcinoma. Int. J. Mol. Sci..

[129] Zam W., Ali L. (2022). Immune Checkpoint Inhibitors in the Treatment of Cancer. Curr. Rev. Clin. Exp. Pharmacol..

[130] Amobi-McCloud A., Muthuswamy R., Battaglia S., Yu H., Liu T., Wang J., Putluri V., Singh P.K., Qian F., Huang R.-Y. (2021). IDO1 Expression in Ovarian Cancer Induces PD-1 in T Cells via Aryl Hydrocarbon Receptor Activation. Front. Immunol..

[131] Crump L.S., Floyd J.L., Kuo L.-W., Post M.D., Bickerdike M., O’Neill K., Sompel K., Jordan K.R., Corr B.R., Marjon N. (2024). Targeting Tryptophan Catabolism in Ovarian Cancer to Attenuate Macrophage Infiltration and PD-L1 Expression. Cancer Res. Commun..

[132] Li Y., Chen K., Chen B., Zeng R., He Y., Wang C., Zhong M., Liu X., Chen X., Xiao L. (2024). Increased Coexpression of PD-L1 and IDO1 is Associated with Poor Overall Survival in Patients with NK/T-Cell Lymphoma. Leukemia.

[133] Löb S., Königsrainer A., Zieker D., Brücher B.L.D.M., Rammensee H.-G., Opelz G., Terness P. (2009). IDO1 and IDO2 are Expressed in Human Tumors: Levo- but Not Dextro-1-Methyl Tryptophan Inhibits Tryptophan Catabolism. Cancer Immunol. Immunother..

[134] Lucarelli G., Rutigliano M., Ferro M., Giglio A., Intini A., Triggiano F., Palazzo S., Gigante M., Castellano G., Ranieri E. (2017). Activation of the Kynurenine Pathway Predicts Poor Outcome in Patients with Clear Cell Renal Cell Carcinoma. Urol. Oncol..

[135] Hornigold N., Dunn K.R., Craven R.A., Zougman A., Trainor S., Shreeve R., Brown J., Sewell H., Shires M., Knowles M. (2020). Dysregulation at Multiple Points of the Kynurenine Pathway is a Ubiquitous Feature of Renal Cancer: Implications for Tumour Immune Evasion. Br. J. Cancer.

[136] Badawy A.A.-B. (2022). Tryptophan Metabolism and Disposition in Cancer Biology and Immunotherapy. Biosci. Rep..

[137] Kennedy B.E., Sharif T., Martell E., Dai C., Kim Y., Lee P.W.K., Gujar S.A. (2016). NAD+ Salvage Pathway in Cancer Metabolism and Therapy. Pharmacol. Res..

[138] Sumitomo M., Takahara K., Zennami K., Nagakawa T., Maeda Y., Shiogama K., Yamamoto Y., Muto Y., Nukaya T., Takenaka M. (2021). Tryptophan 2,3-Dioxygenase in Tumor Cells is Associated with Resistance to Immunotherapy in Renal Cell Carcinoma. Cancer Sci..

[139] Liang X.-H., Chen X.-Y., Yan Y., Cheng A.-Y., Lin J.-Y., Jiang Y.-X., Chen H.-Z., Jin J.-M., Luan X. (2024). Targeting Metabolism to Enhance Immunotherapy within Tumor Microenvironment. Acta Pharmacol. Sin..

[140] Yokota Y., Nozawa H., Sonoda H., Yokoyama Y., Emoto S., Murono K., Sasaki K., Ishihara S. (2024). Indoleamine 2,3-Dioxygenase Inhibitor Suppresses Colon Cancer Cell Migration, Invasion, and Epithelial-Mesenchymal Transition. Anticancer Res..

[141] Peng X., Zhao Z., Liu L., Bai L., Tong R., Yang H., Zhong L. (2022). Targeting Indoleamine Dioxygenase and Tryptophan Dioxygenase in Cancer Immunotherapy: Clinical Progress and Challenges. Drug Des. Devel. Ther..

[142] Sordillo L.A., Sordillo P.P. (2023). Suppression of Kynurenine 3-Monooxygenase as a Treatment for Triple-Negative Breast Carcinoma. Anticancer Res..

[143] León-Letelier R.A., Dou R., Vykoukal J., Sater A.H.A., Ostrin E., Hanash S., Fahrmann J.F. (2023). The Kynurenine Pathway Presents Multi-Faceted Metabolic Vulnerabilities in Cancer. Front. Oncol..

[144] Van Meerhaeghe T., Murakami N., Le Moine A., Brouard S., Sprangers B., Degauque N. (2024). Fine-Tuning Tumor- and Allo-Immunity: Advances in the Use of Immune Checkpoint Inhibitors in Kidney Transplant Recipients. Clin. Kidney J..

[145] Walczak K., Deneka-Hannemann S., Jarosz B., Zgrajka W., Stoma F., Trojanowski T., Turski W.A., Rzeski W. (2014). Kynurenic Acid Inhibits Proliferation and Migration of Human Glioblastoma T98G Cells. Pharmacol. Rep..

[146] Walczak K., Turski W.A., Rajtar G. (2014). Kynurenic Acid Inhibits Colon Cancer Proliferation in Vitro: Effects on Signaling Pathways. Amino Acids.

[147] Walczak K., Zurawska M., Kiś J., Starownik R., Zgrajka W., Bar K., Turski W.A., Rzeski W. (2012). Kynurenic Acid in Human Renal Cell Carcinoma: Its Antiproliferative and Antimigrative Action on Caki-2 Cells. Amino Acids.

[148] Walczak K., Wnorowski A., Turski W.A., Plech T. (2019). Kynurenic Acid and Cancer: Facts and Controversies. Cell. Mol. Life Sci..

[149] Irsik D.L., Chen J.-K., Bollag W.B., Isales C.M. (2024). Chronic Infusion of the Tryptophan Metabolite, Kynurenine Increases Mean Arterial Pressure in Male Sprague Dawley Rats. Am. J. Physiol. Physiol..

[150] Wang H., Wu J., Wei H., Zhang Y., Wang Y., Wang D.W. (2024). Increased Tryptophan Catabolism Provides Predictive Value to Chronic Heart Failure Patients with Low-Grade Inflammation. Inflammation.

[151] Gáspár R., Halmi D., Demján V., Berkecz R., Pipicz M., Csont T. (2021). Kynurenine Pathway Metabolites as Potential Clinical Biomarkers in Coronary Artery Disease. Front. Immunol..

[152] Jasiewicz M., Moniuszko M., Pawlak D., Knapp M., Rusak M., Kazimierczyk R., Musial W.J., Dabrowska M., Kaminski K.A. (2016). Activity of the Kynurenine Pathway and Its Interplay with Immunity in Patients with Pulmonary Arterial Hypertension. Heart.

[153] Fryc J., Naumnik B. (2021). Thrombolome and Its Emerging Role in Chronic Kidney Diseases. Toxins.

[154] El Chamieh C., Larabi I.A., Alencar De Pinho N., Lambert O., Combe C., Fouque D., Frimat L., Jacquelinet C., Laville M., Laville S. (2024). Study of the Association between Serum Levels of Kynurenine and Cardiovascular Outcomes and Overall Mortality in Chronic Kidney Disease. Clin. Kidney J..

[155] Pawlak K., Tankiewicz J., Mysliwiec M., Pawlak D. (2009). Tissue Factor/Its Pathway Inhibitor System and Kynurenines in Chronic Kidney Disease Patients on Conservative Treatment. Blood Coagul. Fibrinolysis.

[156] Pawlak K., Mysliwiec M., Pawlak D. (2009). Hypercoagulability is Independently Associated with Kynurenine Pathway Activation in Dialysed Uraemic Patients. Thromb. Haemost..

[157] Pawlak K., Domaniewski T., Mysliwiec M., Pawlak D. (2009). Kynurenines and Oxidative Status are Independently Associated with Thrombomodulin and von Willebrand Factor Levels in Patients with End-Stage Renal Disease. Thromb. Res..

[158] Wejksza K., Rzeski W., Turski W.A. (2009). Kynurenic Acid Protects against the Homocysteine-Induced Impairment of Endothelial Cells. Pharmacol. Rep..

[159] Pawlak K., Mysliwiec M., Pawlak D. (2012). Hyperhomocysteinemia and the Presence of Cardiovascular Disease are Associated with Kynurenic Acid Levels and Carotid Atherosclerosis in Patients Undergoing Continuous Ambulatory Peritoneal Dialysis. Thromb. Res..

[160] Zapolski T., Kamińska A., Kocki T., Wysokiński A., Urbanska E.M. (2020). Aortic Stiffness-Is Kynurenic Acid a Novel Marker? Cross-Sectional Study in Patients with Persistent Atrial Fibrillation. PLoS ONE.

[161] Balafa O., Fernandez-Fernandez B., Ortiz A., Dounousi E., Ekart R., Ferro C.J., Mark P.B., Valdivielso J.M., Del Vecchio L., Mallamaci F. (2024). Sex Disparities in Mortality and Cardiovascular Outcomes in Chronic Kidney Disease. Clin. Kidney J..

[162] Gamerith G., Mildner F., Merkel P.A., Harris K., Cooney L., Lim N., Spiera R., Seo P., Langford C.A., Hoffman G.S. (2023). Association of Baseline Soluble Immune Checkpoints with the Risk of Relapse in PR3-ANCA Vasculitis Following Induction of Remission. Ann. Rheum. Dis..

[163] Schroecksnadel S., Sucher R., Kurz K., Fuchs D., Brandacher G. (2011). Influence of Immunosuppressive Agents on Tryptophan Degradation and Neopterin Production in Human Peripheral Blood Mononuclear Cells. Transpl. Immunol..

[164] Davis P.M., Nadler S.G., Stetsko D.K., Suchard S.J. (2008). Abatacept Modulates Human Dendritic Cell-Stimulated T-Cell Proliferation and Effector Function Independent of IDO Induction. Clin. Immunol..

[165] Pree I., Bigenzahn S., Fuchs D., Koporc Z., Nierlich P., Winkler C., Brandacher G., Sykes M., Muehlbacher F., Langer F. (2007). CTLA4Ig Promotes the Induction of Hematopoietic Chimerism and Tolerance Independently of Indoleamine-2,3-Dioxygenase. Transplantation.

[166] Boasso A., Herbeuval J.-P., Hardy A.W., Winkler C., Shearer G.M. (2005). Regulation of Indoleamine 2,3-Dioxygenase and Tryptophanyl-TRNA-Synthetase by CTLA-4-Fc in Human CD4+ T Cells. Blood.

[167] Xiao B., Liu B., Song Y., Yu Z., Guo S. (2014). Local Cytotoxic T-Lymphocyte-Associated Antigen-4 Immunoglobulin Inhibition of Rejection Response is Dependent on Indoleamine 2,3-Dioxygenase Activities in the Allograft. Transpl. Proc..

[168] Furuzawa-Carballeda J., Lima G., Uribe-Uribe N., Avila-Casado C., Mancilla E., Morales-Buenrostro L.E., Pérez-Garrido J., Pérez M., Cárdenas G., Llorente L. (2010). High Levels of IDO-Expressing CD16+ Peripheral Cells, and Tregs in Graft Biopsies from Kidney Transplant Recipients under Belatacept Treatment. Transpl. Proc..

[169] Furuzawa-Carballeda J., Lima G., Alberú J., Palafox D., Uribe-Uribe N., Morales-Buenrostro L.E., Reyes Acevedo R., Mondragón G., Chevaile A., Llorente L. (2012). Infiltrating Cellular Pattern in Kidney Graft Biopsies Translates into Forkhead Box Protein 3 Up-Regulation and P16INK4α Senescence Protein down-Regulation in Patients Treated with Belatacept Compared to Cyclosporin A. Clin. Exp. Immunol..

[170] Furuzawa-Carballeda J., Bostock I.C., Lima G., Mancilla-Urrea E., Mondragón G., Reyes-Acevedo R., Chevaile A., Morales-Buenrostro L.E., Llorente L., Alberú J. (2014). Immunophenotyping of Peripheral Immunoregulatory as Well as Th17A and Th22 Cell Subpopulations in Kidney Transplant Recipients under Belatacept or Cyclosporine Treatment. Transpl. Immunol..

[171] Provenzano M., Hu L., Tringali E., Senatore M., Talarico R., Di Dio M., Ruotolo C., La Manna G., Garofalo C., Zaza G. (2024). Improving Kidney Disease Care: One Giant Leap for Nephrology. Biomedicines.

[172] Zakrocka I., Kocki T., Turski W.A. (2017). The Effect of Three Angiotensin-Converting Enzyme Inhibitors on Kynurenic Acid Production in Rat Kidney in Vitro. Pharmacol. Rep..

[173] Zakrocka I., Targowska-Duda K.M., Wnorowski A., Kocki T., Jóźwiak K., Turski W.A. (2019). Angiotensin II Type 1 Receptor Blockers Decrease Kynurenic Acid Production in Rat Kidney in Vitro. Naunyn. Schmiedebergs. Arch. Pharmacol..

[174] Zakrocka I., Załuska W. (2023). The Influence of Cyclooxygenase Inhibitors on Kynurenic Acid Production in Rat Kidney: A Novel Path for Kidney Protection?. Pharmacol. Rep..

[175] Zakrocka I., Kocki T., Urbańska E., Załuska W. (2023). Effects of Fenofibrate and Gemfibrozil on Kynurenic Acid Production in Rat Kidneys In Vitro: Old Drugs, New Properties. Life.

[176] Bednarz K., Kozieł K., Urbańska E.M. (2024). Novel Activity of Oral Hypoglycemic Agents Linked with Decreased Formation of Tryptophan Metabolite, Kynurenic Acid. Life.

[177] Hosseini-Tabatabaei A., Jalili R.B., Khosravi-Maharlooei M., Hartwell R., Kilani R.T., Zhang Y., Ghahary A. (2015). Immunoprotection and Functional Improvement of Allogeneic Islets in Diabetic Mice, Using a Stable Indoleamine 2,3-Dioxygenase Producing Scaffold. Transplantation.

[178] Vavrincova-Yaghi D., Deelman L.E., van Goor H., Seelen M.A., Vavrinec P., Kema I.P., Gomolcak P., Benigni A., Henning R.H., Sandovici M. (2016). Local Gene Therapy with Indoleamine 2,3-Dioxygenase Protects against Development of Transplant Vasculopathy in Chronic Kidney Transplant Dysfunction. Gene Ther..

[179] Colas L., Mongodin E.F., Montassier E., Chesneau M., Guerif P., Hittle L., Giral M., Bromberg J.S., Brouard S. (2020). Unique and Specific Proteobacteria Diversity in Urinary Microbiota of Tolerant Kidney Transplanted Recipients. Am. J. Transpl..

